# Lactate-driven lactylation reprograms chondrocyte fate and joint inflammation in osteoarthritis: linking metabolism with epigenetic regulation

**DOI:** 10.3389/fcell.2026.1939490

**Published:** 2026-08-28

**Authors:** Xinyu Liu, Kangyi Hu, Dengyan Bai

**Affiliations:** 1 Department of Orthopedics, The Second People’s Hospital of Gansu Province, Lanzhou, China; 2 Clinical College of Traditional Chinese Medicine, Gansu University of Chinese Medicine, Lanzhou, China; 3 The Second Clinical Medical College of Northwest Minzu University, Lanzhou, China

**Keywords:** chondrocyte, lactate, lactylation, metabolic reprogramming, osteoarthritis

## Abstract

The sustained progression of osteoarthritis (OA) arises not only from mechanical injury and inflammatory stimulation but also from the gradual loss of chondrocyte homeostatic identity. However, a unifying mechanism explaining how metabolic abnormalities within the joint microenvironment are converted into relatively stable degenerative programs remains lacking. The discovery of lactylation provides a new perspective on this question. OA-associated hypoxia, inflammation, and aberrant mechanical loading can promote glycolytic reprogramming and lactate accumulation, while lactate further channels metabolic stress into chromatin regulation and protein functional networks through histone and non-histone lactylation. Current evidence suggests that this process does not merely amplify a single catabolic pathway, but reshapes chondrocyte fate at multiple levels by impairing matrix-maintaining capacity, driving the transition from a homeostatic phenotype toward catabolic, fibrotic, and senescent states, and altering cellular susceptibility to oxidative injury and pathological cell death. Meanwhile, metabolic reprogramming in synovial cells and immune cells may expand the intra-articular lactate pool, thereby linking intracellular lactylation changes with inter-tissue inflammatory crosstalk. Notably, the effects of lactylation are not uniformly pathogenic, but depend on the modified site, protein substrate, cellular state, redox environment, and disease stage. This bidirectionality is evident in both histone and non-histone lactylation: UGDH K6 lactylation and selected H3K18la-associated programs are linked to matrix damage and fibrotic phenotypes, whereas H3K56la may preserve COL2A1 expression and enhance chondrocyte stress adaptation under specific post-traumatic and redox conditions. Lactylation is therefore better understood as a metabolic state-driven “fate code” rather than a passive marker of lactate accumulation. This review integrates the continuum linking lactate metabolism, site-specific lactylation, and chondrocyte fate remodeling, with particular emphasis on matrix homeostasis, cellular senescence, survival–death transitions, and the inflammatory microenvironment. It also discusses the therapeutic implications of shifting from global modulation of lactylation toward cell- and site-specific intervention. This framework may deepen our understanding of OA chronicity and provide a theoretical basis for metabolic phenotype stratification and disease-modifying therapy.

## 1 Introduction

Osteoarthritis (OA) is a leading musculoskeletal disorder that causes chronic pain, restricted mobility, and impaired quality of life worldwide (Collins et al., 2026; King and Abhishek, 2026). OA was long regarded as a degenerative condition caused by aging and mechanical wear, with its pathology often reduced to structural damage of articular cartilage (Zhang Y. et al., 2026). Growing evidence, however, shows that this wear-and-tear model cannot explain the complex and heterogeneous course of OA (Scerif and Eldridge, 2026). Persistent low-grade inflammation, chondrocyte phenotypic drift, amplification of synovitis, aberrant subchondral bone remodeling, and the imperfect correlation between pain and radiographic damage indicate that OA is not merely a local structural disorder. Rather, it is a disease of the whole joint driven by interacting mechanical, metabolic, inflammatory, and epigenetic processes (Li et al., 2026; Chen et al., 2020). In this revised framework, chondrocytes are not passive end-stage victims of injury. They actively sense microenvironmental stress and reshape their transcriptional fate.

The avascular structure of cartilage exposes chondrocytes to chronically low oxygen and nutrient levels and a limited energy supply (Nordberg et al., 2024). Unlike most cells that depend heavily on mitochondrial oxidative phosphorylation, mature chondrocytes rely substantially on glycolysis even under physiological conditions (Shi and Du, 2024; Zheng et al., 2021). This metabolic feature normally helps them adapt to hypoxia and maintain matrix homeostasis. During OA progression, however, abnormal mechanical loading, inflammatory mediators, oxidative stress, and extracellular matrix damage sustain the glycolytic program (Tan et al., 2022; Wu et al., 2023). Metabolic adaptation then gives way to metabolic dysregulation, characterized by increased glucose uptake, activation of key glycolytic enzymes, and enhanced lactate production and transport (Xu et al., 2025). Lactate may therefore shift from an energy metabolite to a pathological regulator in the OA joint microenvironment.

Lactate in degenerative joint disease was previously viewed mainly as a consequence of local hypoxia and acidification. Its effects were largely confined to disrupted energy metabolism or changes in the inflammatory milieu (Apostolova and Pearce, 2022; Peng et al., 2024). Advances in metabolic epigenetics are changing this view. Lactate is not simply the end product of glycolysis but a metabolic intermediate involved in cell signaling and chromatin regulation (Sun et al., 2017). Histone lactylation is a recently identified epigenetic modification that directly links changes in intracellular lactate to transcriptional control at chromatin (Li et al., 2024a; Yu et al., 2024). Lactate accumulation can promote lysine lactylation and thereby alter chromatin accessibility and transcriptional activity at specific gene promoters (Lv et al., 2023; Zhang D. et al., 2019). Compared with established modifications such as histone acetylation and methylation, lactylation more directly reflects the dynamic influence of cellular metabolism on gene-expression programs (Ziogas et al., 2025). With the discovery of non-histone lactylation, the regulatory scope of this modification has expanded beyond chromatin to include enzyme activity, protein–protein interactions, and subcellular localization. The lactate–lactylation axis therefore provides a new mechanistic entry point for understanding how metabolic abnormalities are converted into persistent changes in cellular phenotype.

Lactylation is not unique to OA. In central nervous system disorders such as neuroinflammation and Alzheimer’s disease, current research has largely focused on metabolic interactions among neurons, microglia, and astrocytes, as well as the regulation of neuroinflammation, synaptic function, and cellular injury by lactylation. In systemic inflammatory and fibrotic diseases, lactylation is more often examined in the context of metabolic reprogramming in immune or stromal cells, hypoxic microenvironments, and pathological tissue remodeling (Tang et al., 2025; Han et al., 2025; Yang et al., 2025). By contrast, the lactate–lactylation axis in OA arises from a distinctive tissue metabolic context. Articular cartilage is avascular, and mature chondrocytes rely heavily on glycolysis even under physiological conditions. Thus, lactate accumulation in OA does not represent a newly acquired metabolic program, but rather a sustained amplification of pre-existing hypoxia-adaptive metabolism under the combined influence of inflammation, aberrant mechanical loading, and matrix injury. In this setting, lactate entering chondrocytes does not produce a uniform biological effect. Instead, it can be translated through histone and non-histone lactylation into distinct, and sometimes opposing, functional outcomes. Lactylation of UDP-glucose dehydrogenase (UGDH) at lysine 6 (K6) and follistatin-like protein 1 (FSTL1)-driven histone H3 lysine 18 lactylation (H3K18la) are associated with impaired matrix synthesis and a fibrotic phenotype, respectively, whereas histone H3 lysine 56 lactylation (H3K56la) can preserve collagen type II alpha 1 chain (COL2A1) expression and enhance chondrocyte stress adaptation under specific post-traumatic and redox conditions, highlighting the marked site-, substrate-, and context dependence of lactylation (Lu et al., 2026; Dong et al., 2026; Di et al., 2026). At the same time, cartilage, synovium, intra-articular immune cells, and subchondral bone coexist within a relatively enclosed joint microenvironment. Lactate produced by different tissues may undergo inter-tissue exchange through diffusion in synovial fluid and monocarboxylate transporter-mediated transmembrane transport and may subsequently be decoded into distinct lactylation outputs in recipient cells. The lactate–lactylation axis in OA should therefore not be regarded as a simple replication within the joint of mechanisms described in other inflammatory or neurodegenerative diseases. Rather, it is shaped by the metabolic constraints of avascular cartilage, site-dependent intracellular lactylation responses, and metabolic interactions across the whole joint.

Against this background, the central question in OA lactylation research is not simply whether lactate levels are elevated, but how sustained lactate pressure becomes consolidated into relatively stable changes in cellular state. Prolonged stress can progressively drive chondrocytes away from their mature homeostatic phenotype, leading to reduced matrix synthesis, enhanced catabolism, hypertrophic or fibrotic phenotypic changes, cellular senescence, and impaired stress recovery (Zheng et al., 2021; Li Z. et al., 2025; Zhu et al., 2023). These alterations cannot be fully explained by transient exposure to a single inflammatory mediator, but are more consistent with sustained cell-fate remodeling under persistent metabolic pressure. Lactate-driven histone and non-histone lactylation provides a direct regulatory interface through which metabolic abnormalities can enter chromatin and protein functional networks, potentially converting transient stress responses into more durable transcriptional and functional states. Meanwhile, matrix fragments and damage-associated signals released from degenerating cartilage can activate synovial cells and intra-articular immune cells, whereas synovium-derived inflammatory mediators can in turn increase the metabolic burden and catabolic activity of chondrocytes. This reciprocal interaction may establish a bidirectional amplification loop among cartilage damage, synovial inflammation, and metabolic reprogramming (Zheng et al., 2021; Adam et al., 2024; Henry Ó and O'Neill, 2025; Kuang et al., 2024). The lactate–lactylation axis may therefore function not merely as an intracellular metabolic response, but as an intermediate mechanism linking tissue injury, inflammatory amplification, and persistent remodeling of cellular states. Accordingly, the value of studying lactylation in OA does not lie in whether this modification is unique to the joint, but in revealing how distinctive tissue architecture, intercellular metabolic exchange, and site-specific regulation jointly shape chondrocyte fate and whole-joint disease progression.

Accordingly, this review re-examines the pathogenesis of OA from the perspective of lactate-driven lactylation. We consider both transcriptional remodeling mediated by histone lactylation and the direct effects of non-histone lactylation on enzyme activity, protein–protein interactions, and subcellular localization. We first summarize the upstream drivers and metabolic basis of enhanced glycolysis and lactate accumulation within the OA joint microenvironment. We then outline the fundamental mechanisms through which lactylation links cellular metabolism to epigenetic regulation, with particular emphasis on its potential roles in chondrocyte fate reprogramming, disruption of matrix homeostasis, cellular senescence, and the persistence of synovial inflammation. By integrating the available evidence, we propose a mechanistic framework in which metabolic stress is translated through lactylation into transcriptional reprogramming and sustained inflammatory degeneration. This framework may provide a new conceptual basis for understanding OA progression and identifying potential targets for disease-modifying therapy.

## 2 Histone lactylation: from lactate metabolism to gene transcription

Lactate was long considered the end product of enhanced glycolysis, with biological roles largely limited to energy metabolism, tissue acidification, and the response to hypoxia (Lawrence, 1953). Metabolic epigenetics now shows that cellular metabolites are more than intermediates or end products of biochemical reactions. They can also act as signals that regulate chromatin and gene transcription (Apostolova and Pearce, 2022; Shi et al., 2024). Metabolites such as acetyl coenzyme A (acetyl-CoA), S-adenosylmethionine, α-ketoglutarate, succinate, and oxidized nicotinamide adenine dinucleotide (NAD^+^) participate in epigenetic control by influencing histone-modifying enzymes or serving directly as modification substrates (Nguyen et al., 2025). This perspective has also changed our understanding of lactate. The discovery of histone lactylation suggests that lactate accumulation not only reflects increased glycolysis, but may also translate cellular metabolism into a transcriptional signal at chromatin (Yu et al., 2024; Chen Y. et al., 2024).

Histone lactylation is an acyl modification of lysine residues, usually denoted as lysine lactylation (Kla) (Zhang D. et al., 2019; Gaffney et al., 2020). When cells become highly glycolytic, rising lactate provides the metabolic basis for transferring lactate-derived acyl groups to histone lysines. This process can alter chromatin structure and transcriptional activity (Gaffney et al., 2020; Bao et al., 2025). Lactate accumulation, however, does not guarantee lactylation. Lactylation may require activated acyl donors such as lactyl coenzyme A (lactyl-CoA) and may involve both enzymatic and nonenzymatic mechanisms (Varner et al., 2020; Zhang X. et al., 2019). The pathways that generate lactyl-CoA, its intracellular availability, substrate selectivity across cell types, and the dose-response relationship between lactate and lactylation remain incompletely defined. Lactylation is therefore best viewed as one route by which lactate metabolism can enter chromatin-regulatory networks, rather than as a simple linear consequence of lactate accumulation.

Among reported histone lactylation sites, histone H3 lysine 18 lactylation (H3K18la), histone H4 lysine 12 lactylation (H4K12la), histone H3 lysine 23 lactylation (H3K23la), and histone H3 lysine 9 lactylation (H3K9la) have received particular attention (Zhang X. et al., 2019). H3K18la was initially used to link lactate accumulation in macrophages with the expression of reparative genes during the later phase of inflammation (Galle et al., 2022; Cai et al., 2024). H4K12la has more recently been associated with cellular senescence, adaptation to stress, and activation of specific transcriptional programs (Li X. et al., 2024; Wang Y. et al., 2024). These sites are generally located in histone tails, where their modification can affect nucleosome conformation, chromatin accessibility, and the recruitment of transcription factors or coactivators (Zhang et al., 2023). Unlike relatively stable epigenetic marks such as DNA methylation, histone lactylation is highly responsive to metabolism. Its dynamic abundance may be influenced by glycolytic activity, lactate production, redox state, and acyl-CoA metabolism (Jiang et al., 2024; Li et al., 2024c). Lactylation may therefore function as a metabolic sensor that converts the current metabolic state of a cell into transcriptional output.

Lactylation is not determined passively by lactate alone. It depends on coordinated writer, eraser, and possibly reader mechanisms. Current evidence suggests that the classical histone acetyltransferases E1A-binding protein p300 and CREB-binding protein (CBP) may also have lactyltransferase activity and use lactyl-CoA to promote lysine lactylation (Xie et al., 2022; Dong et al., 2024; Gonzatti et al., 2025). This possibility is important because p300/CBP are central coactivators that regulate chromatin accessibility and the expression of genes involved in inflammation, differentiation, and stress. If p300/CBP mediate both acetylation and lactylation, lactate accumulation may change their acylation output and reshape transcription at specific loci (Qin et al., 2025; Ju et al., 2024; Gao et al., 2025). Histone deacetylases (HDACs) and sirtuins may remove lactyl groups or indirectly regulate lactylation (Dai et al., 2022; Lv et al., 2025; Moreno-Yruela et al., 2022). Sirtuins are especially sensitive to NAD^+^, while LDHA-dependent conversion between pyruvate and lactate is coupled to the NADH/NAD + cycle (Cantó and Auwerx, 2012; Feron, 2009). Lactate metabolism may thus supply substrates for lactylation and alter the redox state and deacylase activity, indirectly changing the balance between lactylation and other histone modifications.

Lactylation may interact in complex ways with established epigenetic modifications such as acetylation and methylation. Lactylation and acetylation are both lysine acylations. They may compete or cooperate at the same or adjacent lysine residues, thereby affecting local chromatin accessibility, nucleosome stability, and transcriptional complex assembly. Lactate accumulation also occurs in the context of enhanced glycolysis and altered redox balance. This environment can influence lactylation and indirectly reshape acetylation, deacetylation, and methylation by changing acetyl-CoA availability, the NAD^+^/NADH ratio, and mitochondrial metabolism (Zhang D. et al., 2019; Sun et al., 2025). Lactylation is therefore not an isolated additional modification. It is part of a broader epigenetic reorganization caused by metabolic reprogramming. This point is particularly relevant to chronic degenerative disease. Cell fate is rarely determined by one modified site but instead emerges from combined changes in several metabolism-sensitive epigenetic marks that establish a relatively stable transcriptional state.

The function of lactylation is strongly context- and stage-dependent. Early studies implicated lactylation in the expression of repair genes during the later phase of inflammation, suggesting a role in tissue repair and inflammatory resolution. In tumors, fibrosis, immune escape, cellular senescence, and chronic inflammation, however, sustained lactate elevation and excessive lactylation can promote cellular plasticity, proinflammatory gene expression, matrix remodeling, and pathological tissue reorganization (Zhang P. et al., 2026; Ma et al., 2025). Lactylation cannot therefore be classified simply as protective or harmful. Its effect depends on cell type, stimulus duration, lactate source, modification site, target-gene profile, and disease stage. Transient or moderate lactylation may help cells adapt to metabolic stress. Prolonged or uncontrolled lactylation may instead consolidate a temporary stress response into a pathological transcriptional program.

**FIGURE 1:**
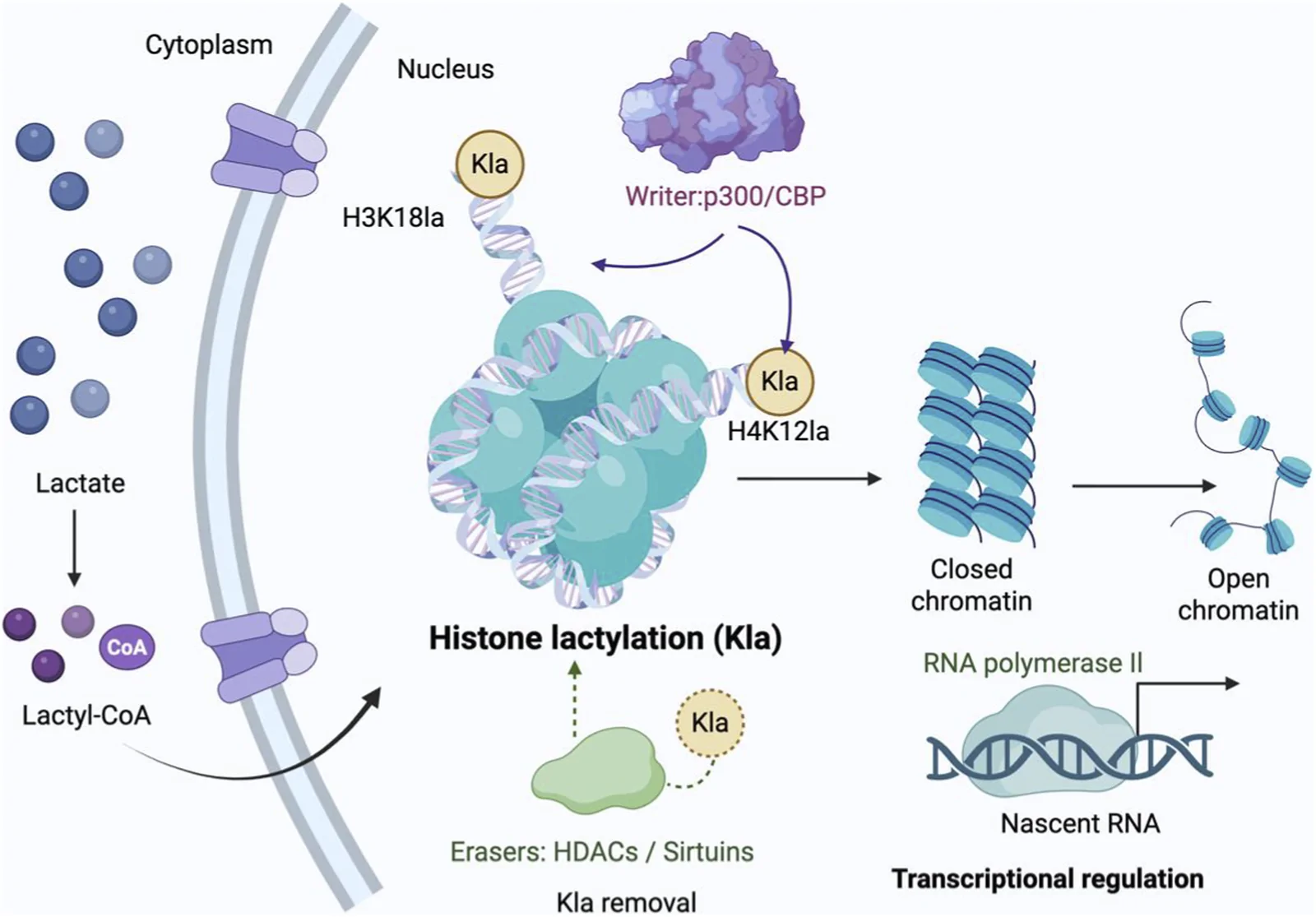
Histone lactylation links lactate metabolism to transcriptional regulation:Intracellular lactate can contribute to the formation of lactylation-related donors, including lactyl-CoA, thereby providing a metabolic basis for histone lysine lactylation (Kla). In the nucleus, p300/CBP acts as a potential writer of histone lactylation, promoting site-specific modifications such as H3K18La and H4K12la, whereas HDACs and sirtuins may function as erasers to remove lactylation marks. Histone lactylation is associated with chromatin remodeling from a relatively closed to an open state, facilitating RNA polymerase II-dependent transcription and thereby linking cellular lactate metabolism to transcriptional regulation. Created with BioRender.com. Publication license number: AH2A3P7FVK.

This context dependence is particularly important in OA. The joint microenvironment is chronically shaped by hypoxia, inflammation, abnormal mechanical stress, and metabolic reprogramming, conditions that favor the induction and persistence of lactylation. As described above, chondrocytes, fibroblast-like synoviocytes, and intra-articular immune cells may all contribute to enhanced glycolysis and lactate accumulation in OA. In this lactate-rich environment, lactylation may be more than a secondary consequence of local metabolic change. It may provide a means of encoding metabolic stress in chromatin. By altering the transcription of genes involved in matrix homeostasis, senescence, autophagic adaptation, inflammatory secretion, and stress repair, lactylation may help shift chondrocytes from a homeostatic to a degenerative phenotype and further remodel synovial inflammation and the joint microenvironment (Xia et al., 2024; Zuo et al., 2026). Histone lactylation thus provides a crucial link between metabolic abnormality and persistent degenerative transcription in OA. Lactate is no longer simply the metabolic endpoint of glycolysis, but an epigenetic signal connecting metabolic reprogramming, chromatin regulation, and cell fate (Figure 1).

## 3 Metabolic reprogramming and lactate accumulation in OA

Articular cartilage has a highly specialized metabolic environment. Mature cartilage contains no blood vessels, nerves, or lymphatic vessels. Oxygen and nutrients are supplied mainly by synovial fluid and limited diffusion through the cartilage matrix. Chondrocytes therefore exist under relatively hypoxic, nutrient-poor conditions with low metabolic turnover (Arai et al., 2024; Zhang et al., 2015). Unlike most cells that rely on mitochondrial oxidative phosphorylation, mature chondrocytes depend heavily on glycolysis under physiological conditions. This dependence supports adaptation to hypoxia and the synthesis and renewal of extracellular matrix. Normal chondrocytes can maintain matrix production under hypoxia and use hypoxia-inducible factor 1α (HIF-1α)-dependent transcription to regulate glucose uptake and glycolysis, thereby preserving survival and function (Zhang J. et al., 2025; Li W. et al., 2025; Du et al., 2025). Glycolysis is therefore not a new abnormality that arises in OA. The critical change is that pathological stimuli amplify an adaptation that normally maintains cartilage homeostasis. Over time, it becomes metabolic reprogramming marked by increased glycolytic flux, lactate production, and altered lactate transport.

Lactate accumulation in OA is not driven by a single stimulus, and the strength of evidence linking individual upstream factors to lactate production is not uniform. Relatively direct evidence indicates that both hypoxia and inflammation can reshape glucose metabolism in chondrocytes. Hypoxia primarily enhances glucose uptake and glycolytic capacity through HIF-1α-dependent transcriptional programs, whereas inflammatory stimulation promotes a further shift from oxidative metabolism toward glycolysis through inflammatory signaling, mitochondrial dysfunction, and oxidative stress. Aberrant mechanical loading can also alter mitochondrial homeostasis and metabolism-related transcriptional states through Piezo channels, integrins, and the yes-associated protein (YAP)/transcriptional coactivator with PDZ-binding motif (TAZ) mechanotransduction network. However, a complete causal chain showing that mechanical loading directly increases lactate production by upregulating glycolytic enzymes and LDHA has not yet been established in OA chondrocytes. When these stimuli ultimately increase glycolytic flux or restrict mitochondrial pyruvate utilization, LDHA occupies the common terminal step that converts pyruvate into lactate. Monocarboxylate transporter (MCT)-mediated lactate export, uptake, and reutilization then determine its distribution within cells, across tissues, and throughout the joint microenvironment. Lactate accumulation in OA should therefore be viewed as the combined result of increased glycolytic carbon flux, restricted mitochondrial pyruvate utilization, and remodeled lactate transport. This metabolic state provides the substrate basis for subsequent histone and non-histone lactylation.

Hypoxia is an intrinsic feature of the articular cartilage microenvironment and provides the physiological basis for the predominant reliance of chondrocytes on glycolysis. Because mature articular cartilage is avascular, oxygen reaches the tissue mainly by diffusion from the synovial fluid and the subchondral bone. Oxygen tension is therefore substantially lower than that in most vascularized tissues and varies across the depth of cartilage. Chondrocytes do not merely tolerate this environment passively. Instead, they establish an adaptive metabolic program through HIF-1α. Under normoxic conditions, HIF-1α undergoes oxygen-dependent hydroxylation, which targets it for rapid recognition and degradation. As oxygen tension falls, this degradation is suppressed. Stabilized HIF-1α accumulates, translocates to the nucleus, heterodimerizes with hypoxia-inducible factor 1β (HIF-1β), and binds hypoxia-response elements to regulate genes involved in glucose uptake and glycolysis. This program increases glucose uptake through glucose transporter 1 (GLUT1) and modulates metabolic enzymes such as hexokinase 2 (HK2), 6-phosphofructo-2-kinase/fructose-2,6-bisphosphatase 3 (PFKFB3), and lactate dehydrogenase A (LDHA), allowing chondrocytes to maintain glycolytic flux and energy production when oxidative phosphorylation is constrained (Xiong et al., 2025; Li J. et al., 2025; Wang X. et al., 2024). The hypoxia–HIF-1α pathway is therefore, in the first instance, a physiological mechanism that supports chondrocyte survival, matrix synthesis, and adaptation to low oxygen, rather than an OA-specific pathogenic pathway.

The critical change in OA is not that chondrocytes become dependent on glycolysis, but that the intensity and duration of this pre-existing adaptive program are altered under persistent pathological stress. Disruption of cartilage architecture, changes in tissue diffusion, increased oxidative stress, and subchondral bone remodeling further disturb local oxygen and nutrient availability. Inflammatory signaling and mitochondrial dysfunction can also intersect with HIF-1α-dependent transcription, sustaining glycolytic dependence beyond its physiological range. In OA cartilage and cytokine-stimulated chondrocyte models, HIF-1α-associated metabolic changes are frequently accompanied by increased glucose uptake, enhanced glycolytic flux, increased LDHA expression, and greater lactate release (He et al., 2025; Wang X. et al., 2025; Sheng et al., 2025). These findings support the involvement of the hypoxia–HIF-1α pathway in lactate production during OA, but its effects should not be reduced to the uniform upregulation of a single glycolytic enzyme. Individual metabolic enzymes may be regulated differently according to cellular state, disease stage, and experimental model. The more consistent feature is that HIF-1α-associated metabolic programs increase chondrocyte reliance on glucose and favor the entry of glycolytic carbon into the pyruvate–lactate pathway.

The contribution of hypoxia to lactate accumulation in OA should therefore be understood as the pathological amplification of a physiological adaptive program, rather than the emergence of an entirely new metabolic pathway. Under physiological hypoxia, appropriately regulated HIF-1α activity supports chondrocyte survival and matrix homeostasis. When hypoxia becomes prolonged and coincides with inflammation, oxidative stress, and impaired mitochondrial pyruvate oxidation, the HIF-1α-dependent glycolytic program may shift from maintaining energy balance to sustaining excessive glucose consumption and lactate output. Under these conditions, more pyruvate is converted into lactate by LDHA. This reaction regenerates NAD^+^, thereby supporting continued glycolysis, while progressively expanding the intracellular and extracellular lactate pools. The sequence of hypoxic sensing, HIF-1α stabilization, activation of the glycolytic program, and LDHA-mediated lactate production thus represents a relatively well-supported upstream metabolic pathway contributing to lactate accumulation in OA. Its pathological consequences nevertheless remain dependent on the duration of activation, mitochondrial metabolic capacity, and the surrounding inflammatory and redox context.

Inflammation represents another important upstream driver of lactate production in OA chondrocytes, but it operates through mechanisms that are distinct from the hypoxia pathway. Whereas hypoxia stabilizes HIF-1α through oxygen-dependent sensing, inflammatory mediators initially act through cytokine receptors and their downstream signaling pathways to reshape both transcriptional and energetic programs. Persistent exposure to interleukin-1β (IL-1β), tumor necrosis factor-α (TNF-α), and other inflammatory mediators in the OA joint activates stress-responsive pathways such as nuclear factor-κB (NF-κB). In addition to inducing matrix metalloproteinases (MMPs), a disintegrin and metalloproteinase with thrombospondin motifs (ADAMTS) family members, and inflammatory mediators, these pathways can upregulate genes involved in glucose metabolism (Chen B. Y. et al., 2024; Arra and Abu-Amer, 2023; Zhuang et al., 2023; Sun et al., 2024). Experimental studies have shown that glycolytic activity increases early after IL-1β stimulation. Moreover, sustained activation of IκB kinase 2 (IKK2, also known as IKKβ)/NF-κB is sufficient to increase the expression of glycolytic genes and LDHA even in the absence of exogenous cytokine stimulation. These findings indicate that inflammatory metabolic changes are not merely secondary consequences of cartilage injury, but form part of an NF-κB-dependent transcriptional program. Chondrocytes isolated from OA cartilage also undergo a metabolic shift from mitochondrial respiration toward glycolysis following IL-1β or TNF-α stimulation, further supporting a direct link between inflammatory signaling and metabolic reprogramming (Arra et al., 2020).

Inflammation also reinforces glycolytic dependence by impairing mitochondrial function. IL-1β can reduce oxygen consumption and the contribution of mitochondrial respiration to energy production, increase the proportion of adenosine triphosphate (ATP) generated through glycolysis, and induce mitochondrial membrane-potential abnormalities and reactive oxygen species (ROS) accumulation. Under these conditions, the capacity of pyruvate to enter the mitochondria and undergo oxidation through the tricarboxylic acid cycle and oxidative phosphorylation is restricted. To preserve short-term ATP production and redox balance, chondrocytes retain a larger proportion of glucose-derived carbon within cytosolic glycolysis. LDHA then reduces pyruvate to lactate while oxidizing NADH to NAD^+^, allowing glycolysis to continue even when oxygen remains available (Zhang X. et al., 2025; Huang et al., 2023; Lan et al., 2025). The inflammatory pathway therefore does not simply reproduce lactate accumulation caused by oxygen deprivation. Rather, it induces a form of aerobic glycolysis jointly driven by inflammatory transcriptional signaling and reduced mitochondrial oxidative capacity. Metabolic-flux studies have shown that IL-1β increases glycolytic proton efflux and suppresses oxidative respiration under oxygen-replete assay conditions, indicating that oxygen availability alone does not determine lactate production during inflammatory stimulation (Ohashi et al., 2021; Wu et al., 2022).

This metabolic shift can, in turn, sustain the inflammatory response. NF-κB activation promotes glycolytic and LDHA-associated reprogramming in chondrocytes, whereas LDHA can stabilize NF-κB zeta (IκB-ζ) through NADH-dependent ROS production, thereby maintaining the expression of inflammatory and catabolic genes such as interleukin-6 (IL-6) and matrix metalloproteinase 13 (MMP13) (Arra et al., 2020). Inflammation and glycolysis may therefore form a positive-feedback loop comprising inflammatory signaling, NF-κB-dependent glycolytic reprogramming, increased LDHA activity and ROS production, and persistence of the inflammatory transcriptional program. HIF-1α may also contribute under inflammatory conditions. Inflammation-associated ROS, metabolic changes, and phosphoinositide 3-kinase (PI3K)/mechanistic target of rapamycin (mTOR) signaling can enhance HIF-1α stability or transcriptional activity even without a marked reduction in oxygen tension, thereby further reinforcing glycolytic adaptation. However, in OA chondrocytes, the complete causal sequence from IL-1β or TNF-α exposure to HIF-1α stabilization, LDHA upregulation, and excessive lactate production is less firmly established than the NF-κB–glycolysis pathway. HIF-1α is therefore better regarded as a cooperating amplifier of inflammation-induced metabolic reprogramming rather than its sole or fully established mediator.

The inflammatory pathway can thus be divided into two coupled mechanistic branches. First, IL-1β and TNF-α directly reprogram glycolytic gene expression through NF-κB and related signaling pathways, increasing glucose utilization and LDHA expression. Second, inflammation-induced mitochondrial dysfunction and ROS accumulation restrict pyruvate oxidation, making chondrocytes increasingly dependent on glycolysis for energy production and NAD^+^ regeneration. Together, these processes divert more pyruvate toward lactate and maintain high glycolytic activity and lactate output even under relatively normoxic conditions. Inflammation-induced lactate accumulation is therefore not simply an extension of the hypoxia response, but the product of inflammatory signaling, restricted mitochondrial metabolism, and an LDHA-associated metabolic–inflammatory feedback loop.

Aberrant mechanical loading is also an important upstream contributor to metabolic reprogramming in OA, although its connection to lactate production is more indirect than that of hypoxia or inflammation. Physiological levels of cyclic compression and shear support chondrocyte differentiation, matrix synthesis, and energy homeostasis. When the magnitude, duration, or spatial distribution of mechanical loading exceeds the adaptive capacity of cartilage, however, mechanical signals are converted from homeostatic cues into damaging stimuli. Excessive compression, abnormal shear stress, and joint malalignment not only disrupt the extracellular matrix directly but also alter mechanosensing, cytoskeletal tension, and organelle homeostasis in chondrocytes, progressively favoring inflammatory, oxidative, and catabolic states (Wang et al., 2022; Shao et al., 2024). The influence of mechanical stress on lactate metabolism therefore cannot be explained solely by increased energy demand. It also reflects impaired intracellular decoding of mechanical signals and a declining capacity to maintain metabolic balance.

At the plasma membrane and cytoskeletal interface, integrin–focal adhesion complexes and mechanosensitive ion channels such as Piezo provide major entry points for sensing abnormal loading. Mechanical overload can activate piezo-type mechanosensitive ion channel component 1 (Piezo1), induce sustained Ca^2+^ influx, and subsequently promote mitochondrial Ca^2+^ overload, ROS accumulation, and lipid peroxidation, thereby increasing susceptibility to ferroptosis. Mechanical overload can also induce chondrocyte senescence by disrupting mitochondrial function and energy homeostasis, demonstrating that membrane-level mechanical signals can propagate to mitochondrial metabolic systems. Integrins and their associated focal-adhesion and cytoskeletal networks sense changes in matrix stiffness and tissue tension and regulate the subcellular localization and transcriptional activity of mechanotransducers such as YAP and TAZ. Studies in OA models have shown that integrin/YAP signaling contributes to mechanically induced chondrocyte injury and ferroptosis, whereas transcriptomic analyses indicate that mechanical stress reshapes immune- and metabolism-related gene networks in chondrocytes (Zhang et al., 2022; Wang S. et al., 2025). Together, these findings support a mechanistic link involving abnormal mechanosensing, altered Ca^2+^ and cytoskeletal signaling, mitochondrial dysfunction, and metabolism-related transcriptional remodeling.

Nevertheless, current evidence in OA chondrocytes does not establish that Piezo or integrin activation necessarily drives the nuclear translocation of YAP/TAZ and directly induces specific glycolytic enzymes or LDHA. A more plausible interpretation is that abnormal mechanical loading promotes a glycolytic shift through two interconnected routes. First, Piezo-mediated Ca^2+^ dysregulation and mitochondrial injury may restrict pyruvate entry into the tricarboxylic acid cycle and reduce its utilization through oxidative phosphorylation, thereby increasing reliance on cytosolic glycolysis as a compensatory energy source. Second, the integrin–cytoskeleton–YAP/TAZ transcriptional network may alter the expression of metabolic and stress-response genes and may increase glucose utilization and glycolytic capacity. Both routes could ultimately increase the glycolytic carbon entering the pyruvate–lactate reaction. However, direct transcriptional regulation of LDHA by YAP/TAZ should currently be regarded as a mechanistic link that remains to be tested, rather than an established causal pathway in OA chondrocytes.

Against a background of sustained glycolytic activity, LDHA serves as the key enzymatic step that converts metabolic reprogramming into increased lactate production. LDHA catalyzes the reduction of pyruvate to lactate and supports continued glycolysis through the NADH/NAD^+^ cycle (Sharma et al., 2022). In OA-related studies, increased LDHA expression is frequently associated with elevated lactate production and a degenerative chondrocyte phenotype, whereas LDHA inhibition or reduction of lactate levels can attenuate at least part of the catabolic response to inflammatory stimulation (Lu et al., 2026; Arra et al., 2020; Gao et al., 2024). Hypoxia, inflammation, and abnormal mechanical loading arise from distinct upstream signals. However, when these stimuli increase glycolytic carbon flux or restrict mitochondrial pyruvate utilization, more pyruvate becomes available for LDHA-mediated conversion into lactate. LDHA therefore occupies the terminal reaction through which multiple OA-associated stresses can converge on lactate production. Its role is not simply a passive consequence of enhanced glycolysis, but a key metabolic step that translates upstream stress into increased lactate output.

At the same time, LDHA should not be regarded as the sole rate-limiting determinant of lactate levels in OA. Lactate burden is also influenced by glucose uptake, upstream glycolytic enzymes such as HK2 and PFKFB3, mitochondrial pyruvate utilization, lactate dehydrogenase B (LDHB) -mediated lactate reutilization, and transmembrane lactate transport. LDHA is therefore more accurately described as a shared terminal convergence point than as the only metabolic bottleneck controlling lactate accumulation.

Lactate accumulation also depends on transmembrane transport and local clearance. Monocarboxylate transporters (MCTs) control lactate movement across the cell membrane. Monocarboxylate transporter 4 (MCT4) primarily mediates lactate export in highly glycolytic cells, whereas monocarboxylate transporter 1 (MCT1) contributes to lactate uptake and reuse (Tassinari et al., 2025; Kobayashi et al., 2021). Hypoxia, inflammation, or increased metabolic demand can adaptively alter MCT1 and MCT4 expression and function, changing the distribution of lactate inside and outside cells (Kobayashi et al., 2021). Elevated lactate in the OA joint may therefore reflect not only increased production, but also imbalances in export, reuptake, and local diffusion. As chondrocyte glycolysis increases, MCT-dependent transport may alter lactate gradients among cartilage, synovium, and the joint cavity. Lactate can then shift from an intracellular end product to an exchangeable metabolic signal within the joint microenvironment (Martin et al., 2025).

Synovial tissue and intra-articular immune cells may also contribute to the local lactate pool in OA. Although OA synovitis is usually chronic and low grade, activated fibroblast-like synoviocytes and macrophages can become more glycolytic and increase the release of lactate and inflammatory mediators (Wu et al., 2025; Huang et al., 2024). Lactate from these cells adds to the metabolic burden of the joint cavity. Cytokines released by inflamed synovium can in turn drive chondrocytes toward glycolysis. Lactate accumulation in OA is therefore not confined to chondrocytes. It may arise from local metabolic remodeling shared by cartilage, synovium, and immune cells.

**FIGURE 2:**
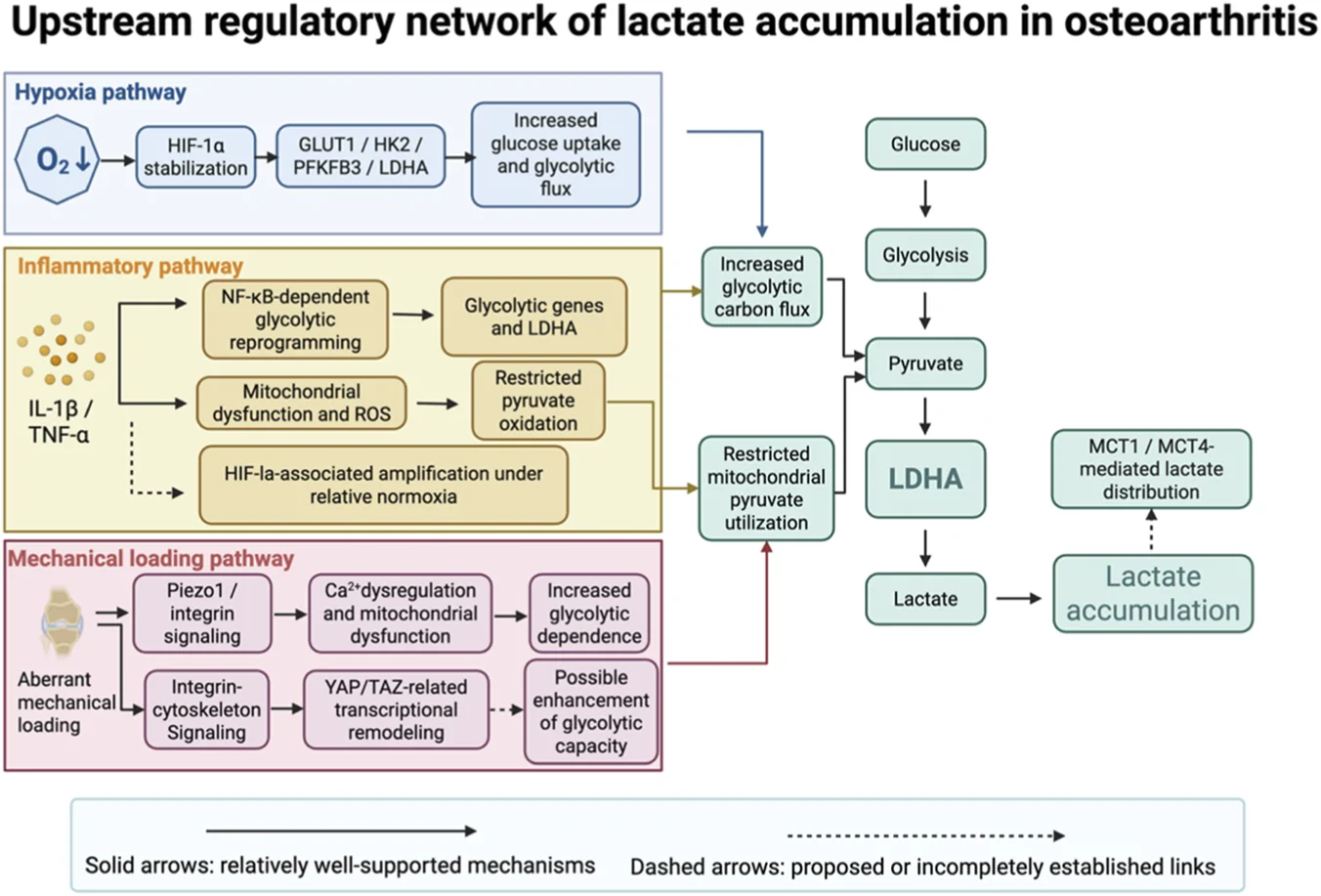
Upstream regulatory network of lactate accumulation in osteoarthritis. Hypoxia, inflammatory cytokines, and aberrant mechanical loading promote lactate accumulation in osteoarthritic chondrocytes through distinct but interconnected pathways. Hypoxia stabilizes HIF-1α and enhances the expression of GLUT1, HK2, PFKFB3, and LDHA, thereby increasing glucose uptake and glycolytic carbon flux. IL-1β and TNF-α promote NF-κB-dependent glycolytic reprogramming and impair mitochondrial function, leading to ROS accumulation, restricted pyruvate oxidation, and sustained glycolysis even under relatively normoxic conditions. HIF-1α may further amplify this inflammation-associated metabolic response. Aberrant mechanical loading activates Piezo1-and integrin-associated signaling, resulting in Ca^2+^ dysregulation, mitochondrial dysfunction, and increased glycolytic dependence. Integrin–cytoskeleton–YAP/TAZ signaling may also enhance glycolytic capacity, although this mechanistic link remains incompletely established in OA chondrocytes. These upstream pathways converge by increasing glycolytic carbon flux or restricting mitochondrial pyruvate utilization, thereby favoring LDHA-mediated conversion of pyruvate to lactate. MCT1 and MCT4 further regulate lactate distribution across the plasma membrane and contribute to the formation of a lactate-rich joint microenvironment. Solid arrows indicate relatively well-supported mechanisms, whereas dashed arrows denote proposed or incompletely established links. Created with BioRender.com. Publication license number: YS2A3P8HNI.

Overall, lactate accumulation in OA reflects the pathological amplification of the intrinsic glycolytic dependence of chondrocytes under persistent stress. Hypoxia enhances glucose uptake, glycolytic gene expression, and LDHA-mediated lactate production through HIF-1α-dependent programs. Inflammation sustains a highly glycolytic state even under relatively normoxic conditions by impairing mitochondrial function, increasing ROS production, and activating NF-κB/MAPK and HIF-1α-associated signaling. Aberrant mechanical loading further alters glucose metabolism through integrin-, Piezo-, cytoskeleton-, and YAP/TAZ-related mechanotransduction networks that intersect with hypoxic and inflammatory pathways. When these upstream inputs increase glycolytic carbon flux or restrict mitochondrial pyruvate utilization, they converge functionally at the LDHA-catalyzed conversion of pyruvate to lactate, whereas MCT1 and MCT4 determine lactate distribution within cartilage and across the whole-joint microenvironment. The resulting lactate-rich state is therefore both a consequence of disrupted metabolic homeostasis in OA and an essential metabolic basis for lactate signaling and subsequent histone and non-histone lactylation (Figure 2).

## 4 Lactylation-dependent remodeling of chondrocyte phenotype and fate

Mature chondrocytes preserve the structural and mechanical properties of articular cartilage by maintaining a SRY-box transcription factor 9 (SOX9) -driven differentiation program and continuously producing type II collagen and aggrecan. In OA, chondrocytes gradually lose this homeostatic phenotype. Matrix synthesis declines, catabolism increases, fibrotic or hypertrophy-like differentiation emerges, and cellular senescence and pathological cell death become more frequent. These changes interact but do not follow a strict sequence. Together, they reflect a progressive loss of phenotypic stability and stress-adaptation capacity. Lactate accumulation and lactylation provide a new metabolic perspective on these changes. Histone lactylation can alter transcription at specific loci and regulate programs related to glycolysis, fibrosis, senescence, and lipid peroxidation. Nonhistone lactylation can directly affect enzyme activity, protein interactions, and subcellular localization. Lactylation therefore influences chondrocytes through both transcriptional changes and direct regulation of key proteins (Zhang D. et al., 2019). Lactylation-mediated reprogramming of chondrocyte fate is not simply the upregulation of one degenerative gene. It is a multilayered remodeling of chondrocyte identity, function, and adaptability as metabolic stress enters both chromatin-regulatory and protein-functional networks.

### 4.1 Lactylation and the loss of the matrix-maintaining phenotype

The earliest sign of altered chondrocyte fate may be an imbalance between anabolism and catabolism. Under physiological conditions, SOX9 maintains the expression of cartilage-specific genes such as collagen type II alpha one chain (COL2A1) and aggrecan (ACAN) and preserves the hyaline cartilage phenotype. As OA progresses, this program weakens, whereas matrix metalloproteinase 13 (MMP13), ADAMTS family members, runt-related transcription factor 2 (RUNX2), collagen type X alpha one chain (COL10A1), and fibrosis-related genes increase (Glasson et al., 2005; Little et al., 2009). Current evidence does not establish all of these molecules as direct transcriptional targets of lactylation. Lactylation is more likely to disrupt matrix homeostasis in two complementary ways. It may directly impair proteins required for matrix synthesis and may also rewrite the chondrocyte phenotypic program at the chromatin level.

Direct evidence for the first mechanism comes from nonhistone lactylation of UGDH. UGDH generates UDP-glucuronic acid for glycosaminoglycan synthesis and is essential for maintaining cartilage proteoglycans (Wen et al., 2014; Clarkin et al., 2011). Lan et al. found that lactate accumulation and global protein lactylation increased together in synovial fluid from patients with OA, primary chondrocytes, and cartilage from mice subjected to destabilization of the medial meniscus (DMM). Lactate or IL-1β further promoted extracellular matrix degradation, whereas reducing lactate production or inhibiting p300 improved this phenotype. Lactylome profiling identified marked lactylation of UGDH at K6. This modification reduced UGDH activity and directly limited glycosaminoglycan synthesis. It also changed the nucleocytoplasmic distribution of UGDH and weakened its interaction with signal transducer and activator of transcription 1 (STAT1), relieving repression of mitogen-activated protein kinase kinase 8 (MAP3K8) transcription and activating catabolic MAPK signaling. The K6R mutation or p300 inhibition restored UGDH function and reduced cartilage damage. Lactylation can therefore disrupt cartilage homeostasis by reducing matrix synthesis and increasing matrix degradation (Lan et al., 2025). This finding extends lactylation beyond conventional transcriptional regulation. Even before chondrocytes fully lose cartilage-specific gene expression, lactylation of a key protein may impair their metabolic capacity to maintain the matrix.

The second mechanism involves direct rewriting of cell identity by histone lactylation. Follistatin-like protein 1 (FSTL1) is a secreted glycoprotein associated with inflammatory arthritis and has seemingly opposing effects. It aggravates chondrocyte apoptosis and inflammatory signaling but also promotes proliferation and fibrosis (Xu et al., 2020; Li et al., 2021; Grandi et al., 2020). Lu et al. found that FSTL1 upregulated HK2, pyruvate kinase M(PKM), and LDHA through a HIF-1-related metabolic program, resulting in enhanced glycolysis, lactate accumulation, and increased H3K18la in chondrocytes. RNA sequencing, H3K18la cleavage under targets and tagmentation (CUT&Tag), and chromatin immunoprecipitation (ChIP) analyses showed enrichment of H3K18la at fibrosis-related loci, including Itga6, Cxcl10, and Parp16, and increased transcription of these genes. Reducing lactate production or inhibiting PI3K/mTOR signaling lowered H3K18la and fibrotic markers. Exogenous lactate partially restored the phenotype. Manipulating FSTL1 in DMM mice also altered cartilage fibrosis and disease severity (Lu et al., 2026). Lactylation therefore causes more than a loss of matrix. It changes chondrocyte identity by shifting mature cells that maintain hyaline cartilage toward a fibrocartilage-like transcriptional state.

During this transition, a metabolic positive-feedback loop may allow lactylation to sustain the catabolic advantage. Xia et al. observed concurrent increases in LDHA, lactate, and H3K18la in cellular and animal models of OA. LDHA knockdown reduced glycolytic activity, MMP13 expression, and cartilage damage. ChIP and reporter assays further suggested that LDHA-dependent H3K18la enhanced triosephosphate isomerase 1 (TPI1) transcription. Interference with TPI1-related sites reduced glucose consumption and lactate production and partially restored COL2 expression (Xia et al., 2024). The hierarchy between the TPI1 protein-site mutation and promoter H3K18la requires clarification. Nevertheless, the findings suggest that lactylation may maintain lactate production by upregulating a glycolytic enzyme. This creates a positive-feedback loop in which increased glycolysis drives lactate accumulation and H3K18la, which then further strengthens glycolysis. A metabolic shift initially induced by hypoxia or inflammation may therefore persist after the stimulus subsides and become part of the catabolic chondrocyte phenotype.

Lactylation-mediated loss of matrix homeostasis cannot be reduced to increased MMP13 or ADAMTS5. UGDH K6 lactylation impairs the capacity of chondrocytes to produce matrix (Lan et al., 2025). H3K18la-associated transcriptional remodeling promotes a fibrotic and catabolic phenotype (Lu et al., 2026). Positive feedback between lactylation and glycolysis provides sustained metabolic support for this state. Together, these processes turn chondrocytes from matrix maintainers into matrix consumers. Whether this phenotypic drift becomes difficult to reverse may depend on the ability of lactylation to consolidate transient stress into cellular senescence and a persistent senescence-associated secretory phenotype (SASP) (Zuo et al., 2026).

### 4.2 Lactylation-driven chondrocyte senescence and SASP

Chondrocyte senescence is not simply proliferative arrest. It is a stable cellular state accompanied by metabolic remodeling, chromatin alterations, and reprogrammed secretory activity (Calcinotto et al., 2019; Hernandez-Segura et al., 2018). Senescent chondrocytes typically show increased cyclin-dependent kinase inhibitor 2A (p16^INK4a), cyclin-dependent kinase inhibitor 1A (p21), phosphorylated histone H2AX (γ-H2AX), and senescence-associated β-galactosidase (SA-β-gal), while releasing SASP components such as IL-6, IL-8, chemokines, and matrix metalloproteinases (Ji et al., 2022; Fu et al., 2024). Senescence is therefore not a passive outcome of matrix damage. Once established, senescent cells can continuously release inflammatory and catabolic mediators that injure neighboring chondrocytes. A cell-autonomous change in fate can thus expand into a degenerative tissue environment.

Zuo et al. provided the most complete direct evidence to date that lactate accumulation can drive a senescent fate. In clinical OA cartilage, animal models, and IL-1β-stimulated chondrocytes, enhanced glycolysis and lactate accumulation coincided with increases in SA-β-gal, p16, p21, γ-H2AX, and SASP. Exogenous lactate aggravated these features, whereas inhibition of glycolysis or lactate production had the opposite effect. Comparison of several histone sites identified H4K12la as the most prominently altered lactylation mark. Integrated H4K12la CUT&Tag and transcriptomic analyses then identified tripartite motif-containing protein 29 (TRIM29) as a key target. ChIP confirmed enrichment of H4K12la at the TRIM29 regulatory region. TRIM29 knockdown attenuated lactate-induced senescence and SASP, whereas TRIM29 overexpression restored the phenotype (Zuo et al., 2026). This evidence shows that lactate can encode metabolic information in a senescence-related transcriptional program through H4K12la, rather than promoting senescence only indirectly through acidification or oxidative stress (Zuo et al., 2026). The mechanism changes not only the abundance of senescence markers, but also how chondrocytes respond to stimulation. Transient inflammation is often partly reversible. Once H4K12la sustains TRIM29 expression, however, chondrocytes may not fully return to homeostasis when the external stimulus wanes. Mechanistic intervention in the same study further supported the lactate–H4K12la–TRIM29 pathway. Sirtuin 1 (SIRT1), an NAD^+^-dependent deacylase with reported delactylase activity, was reduced in OA cartilage. In chondrocytes exposed to high lactate, SIRT1 overexpression decreased H4K12la and TRIM29 expression, suppressed downstream PI3K–AKT activation, and attenuated senescence-associated changes, including SA-β-gal activity, γ-H2AX accumulation, and SASP production. Conversely, SIRT1 knockdown enhanced these responses. In the rat OA model, intra-articular administration of SRT1720, a pharmacological SIRT1 activator, similarly reduced cartilage H4K12la, p16, and p21. A complementary intervention targeted lactate production upstream. Oxamate, an inhibitor of LDHA-mediated pyruvate-to-lactate conversion, reduced lactate accumulation, PanKla and H4K12la levels, and chondrocyte senescence both *in vitro* and following intra-articular administration in the OA model. These interventions link reduced lactate availability or enhanced delactylation with suppression of the H4K12la–TRIM29 senescence program and improvement of cartilage pathology (Zuo et al., 2026).

Earlier senescence studies provide mechanistic support for this upstream role of lactylation. Philipot et al. found that increased p16^INK4a in human OA cartilage and IL-1β-stimulated primary chondrocytes coincided with increased IL-6, IL-8, MMP1, and MMP13. Overexpression of p16^INK4a alone induced MMP1 and MMP13, showing that the senescence program can actively damage matrix rather than merely mark degenerated cartilage (Philipot et al., 2014). Xie et al. used chondrocyte-specific Pten deletion to produce sustained Akt activation. The mice developed ROS accumulation, chondrocyte senescence, increased SASP, and spontaneous OA. Restricting Akt signaling or reducing oxidative stress with N-acetylcysteine attenuated senescence and cartilage damage (Xie et al., 2019). These studies did not directly measure lactylation, but they show that sustained Akt activation, oxidative stress, and senescence downstream of the H4K12la-TRIM29 axis are sufficient to drive disease.

Experiments that eliminate senescent cells further confirm that this cell fate helps maintain cartilage degeneration. Ro et al. identified dipeptidyl peptidase-4 (DPP-4)-positive senescent chondrocytes in human OA cartilage with high SA-β-gal, p16, p21, and SASP expression. Selective depletion of these cells reduced inflammatory and senescence markers and improved both the cartilage matrix phenotype and experimental OA (Jeon et al., 2017; Ro et al., 2024; Maurer et al., 2025). Together with the H4K12la findings, these results support a continuous sequence: lactate accumulation promotes chondrocyte senescence through site-specific lactylation, and SASP from senescent cells amplifies this cell-autonomous change into matrix degradation and paracrine inflammation.

IL-6, chemokines, and matrix-degrading enzymes released as part of the SASP act on more than the extracellular matrix. They may also increase the inflammatory and metabolic burden on neighboring chondrocytes and thereby promote glycolysis and lactate production (Jeon et al., 2019). Lactylation-driven senescence may therefore form another positive-feedback loop. Lactate induces lactylation, lactylation consolidates the senescence program, and SASP promotes further lactate accumulation through inflammation and metabolic stress. Lactylation may act not only as an upstream trigger of senescence, but also as an epigenetic fulcrum that allows senescence to spread and persist in cartilage. The stability of this fate, however, also depends on whether chondrocytes retain the capacity to remove damaged organelles, restore energy homeostasis, and terminate the stress response.

### 4.3 Site-specific lactylation in chondrocyte survival and death

Unlike cellular senescence, pathological cell death permanently removes chondrocytes from the tissue homeostatic network. Mature articular cartilage has limited cell turnover and regenerative capacity. Chondrocyte death therefore reduces matrix synthesis directly and releases damage-associated molecules that increase inflammatory and oxidative stress in neighboring cells (Newton et al., 2024; Green, 2024). OA progression depends not only on whether a particular death pathway is activated, but also on whether persistent mechanical, inflammatory, and metabolic stress resets the survival-death threshold of chondrocytes. Current evidence suggests that lactylation may alter this threshold by regulating lipid peroxidation, matrix metabolism, and adaptation to oxidative stress. Its effect is not uniformly pro-death, but depends strongly on the modified site, protein substrate, and microenvironment.

Ferroptosis provides the most direct evidence that lactylation can influence chondrocyte death in OA. Ferroptosis is driven by iron-dependent lipid peroxidation and reflects the balance between lipid oxidation and cellular antioxidant defense (Dixon and Olzmann, 2024). Glutathione peroxidase 4 (GPX4) detoxifies phospholipid hydroperoxides, whereas solute carrier family seven member 11 (SLC7A11), a component of the cystine/glutamate antiporter system Xc^−^, supports glutathione synthesis and ferroptosis resistance. By contrast, acyl-CoA synthetase long-chain family member 4 (ACSL4) promotes the incorporation of polyunsaturated fatty acids into membrane phospholipids and increases the pool of oxidizable lipid substrates. Damaged regions of human OA cartilage show reduced GPX4 and SLC7A11, increased ACSL4, and accumulation of lipid peroxides and malondialdehyde. These abnormalities become more pronounced with cartilage degeneration. In chondrocytes derived from relatively mild OA cartilage, ferrostatin-1 restored GPX4 and SLC7A11, reduced ACSL4 expression and lipid peroxidation, and improved COL2A1 and MMP13 expression (Xu et al., 2023; Liu Y. et al., 2024). Zhang et al. further placed lactylation upstream of this ferroptotic program. Lactate dehydrogenase B (LDHB), an enzyme that catalyzes the reversible conversion between lactate and pyruvate, increased together with H3K18la, Fe^2+^, and lipid ROS in cartilage from OA mice and in IL-1β-stimulated chondrocytes. LDHB knockdown or pharmacological LDH inhibition reduced H3K18la, ACSL4 expression, iron accumulation, and lipid peroxidation, whereas lactate supplementation reversed these effects. ChIP and reporter assays suggested that H3K18la enrichment at the ACSL4 regulatory region enhanced ACSL4 transcription, while ACSL4 overexpression counteracted the reduction in ferroptotic features produced by LDHB knockdown (Zhang Y. et al., 2025). Lactylation may thus increase sensitivity to oxidative stress by expanding the pool of oxidizable lipids. This evidence still requires caution. Ferroptosis was assessed mainly through Fe2+, lipid ROS, ACSL4, and cell viability. Although ACSL4 overexpression provided functional rescue, validation with chondrocyte-specific ACSL4 models and several ferroptosis inhibitors is lacking. Moreover, LDHB normally catalyzes the reversible conversion of lactate to pyruvate, so its net effect on the lactate pool and lactylation may differ across redox states.

The regulation of apoptosis by lactylation is more complex. As noted above, UGDH K6 lactylation impairs glycosaminoglycan synthesis and is accompanied by increased apoptosis. This modification reduces UGDH activity, suppresses glycosaminoglycan synthesis, and alters the nucleocytoplasmic distribution of UGDH and its interaction with STAT1. MAP3K8 transcription and MAPK signaling consequently increase. Blocking lactate production, inhibiting p300, or introducing the K6R mutation attenuates matrix damage and apoptosis-related changes (Lan et al., 2025). Nonhistone lactylation may therefore lower the apoptotic threshold by simultaneously disrupting matrix synthesis and survival signaling. UGDH lactylation does more than reduce the activity of one metabolic enzyme. It weakens the ability of chondrocytes to maintain both themselves and their surrounding matrix.

Lactylation does not invariably promote chondrocyte death. In a study of post-traumatic OA, Dong et al. found that oxidative stress reduced nuclear lactylation in chondrocytes. A relatively reduced state induced by α-ketoglutarate promoted lactate uptake, restored H3K56la, and markedly reduced oxidative stress-induced apoptosis. Mechanistically, increased H3K56la promoted HIF-1α expression. HIF-1α then bound the Col2a1 promoter and enhanced type II collagen transcription. Inhibition of glycolysis, LDHA, or p300/CBP reduced H3K56la and COL2A1, whereas lactate supplementation or restoration of HIF-1α partly reversed these changes. Activation of this pathway also improved cartilage pathology in an anterior cruciate ligament transection model of post-traumatic OA (Dong et al., 2026). In chondrocytes that remain capable of repair under relatively reducing conditions, lactate is therefore not merely a harmful metabolite. Through the H3K56la-HIF-1α-COL2A1 axis, it can support matrix synthesis and resistance to apoptosis.

The pro-ferroptotic H3K18la–ACSL4 axis and the anti-apoptotic H3K56la–HIF-1α–COL2A1 axis appear to produce opposing outcomes, but together they illustrate the site- and context-dependent nature of lactylation in chondrocyte fate (Dong et al., 2026; Zhang Y. et al., 2025). H3K18la can direct lactate-derived signals toward ACSL4-dependent lipid peroxidation and thereby increase ferroptotic susceptibility UGDH K6 lactylation (Zhang Y. et al., 2025). By contrast, disrupts matrix metabolism and is associated with apoptosis-related changes (Lan et al., 2025). Under a specific post-traumatic and reducing environment, H3K56la enhances HIF-1α-dependent COL2A1 transcription and protects chondrocytes against oxidative stress-induced apoptosis (Dong et al., 2026). Global lactylation levels are therefore insufficient to predict the survival outcome of chondrocytes. The biological effect depends on the modified site, downstream effector pathway, cellular redox state, and disease context. This dependence may also vary with OA etiology and stage. Inflammation-driven, age-related, and post-traumatic OA differ in lactate source, oxidative stress, and chromatin state. Consistent with this interpretation, inhibition of ferroptosis appears to protect human OA cartilage more effectively at relatively early stages (Xu et al., 2023).

**FIGURE 3:**
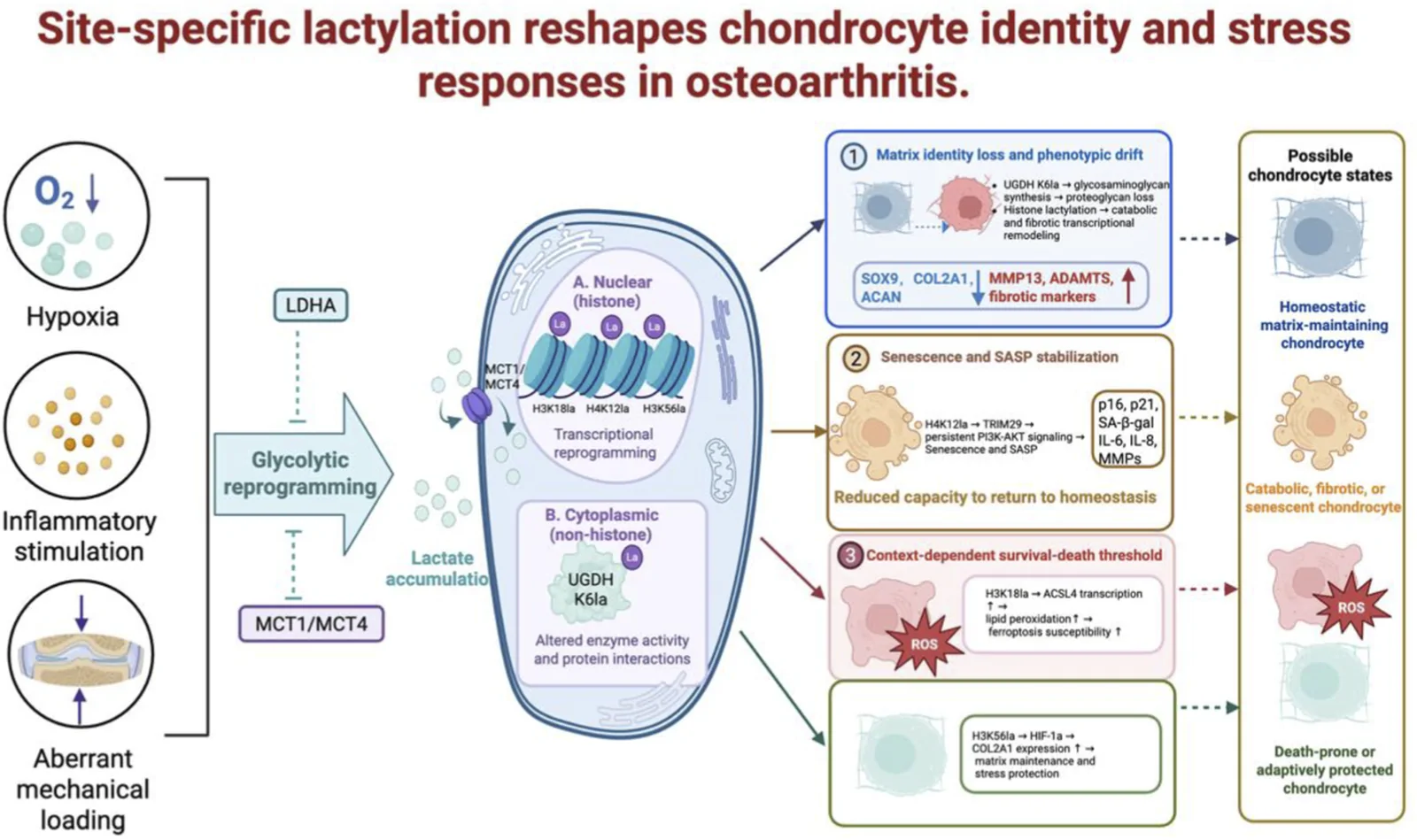
Site-specific lactylation reshapes chondrocyte identity and stress responses in osteoarthritis:Hypoxia, inflammatory stimulation, and aberrant mechanical loading promote glycolytic reprogramming and lactate accumulation in osteoarthritic chondrocytes, with LDHA and monocarboxylate transporters contributing to lactate production and transport. Increased lactate availability is linked to both nuclear histone lactylation and cytoplasmic non-histone lactylation. Site-specific histone lactylation, including H3K18la, H4K12la, and H3K56la, may induce distinct transcriptional programs involved in matrix loss, senescence-associated secretory phenotype stabilization, and context-dependent stress adaptation or cell-death susceptibility. Meanwhile, non-histone lactylation, exemplified by UGDH K6 lactylation, can alter enzyme activity and protein interactions, thereby disrupting extracellular matrix homeostasis. Together, these lactylation-dependent regulatory events may drive chondrocyte phenotypic drift from a homeostatic matrix-maintaining state toward catabolic, fibrotic, senescent, death-prone, or adaptively protected states, highlighting the site- and context-dependent effects of lactylation in osteoarthritis. Created with BioRender.com. Publication license number: EZ2A3P801Q.

In summary, lactylation does not determine a single universal mode of chondrocyte death. Instead, site-specific lactylation changes the threshold for distinct injury programs. The H3K18la–ACSL4 pathway primarily increases susceptibility to ferroptosis by enhancing lipid-peroxidation machinery. UGDH K6 lactylation is associated with matrix failure and apoptosis-related injury, whereas the H3K56la–HIF-1α–COL2A1 pathway reduces oxidative stress-induced apoptosis under a specific post-traumatic and reducing context. Ferroptosis and apoptosis should therefore not be treated as interchangeable outcomes. The predominant response depends on the modified site, downstream effector network, redox environment, and OA phenotype or stage (Figure 3).

### 4.4 Bidirectional and site-specific regulation of chondrocyte fate by lactylation in OA

Collectively, current evidence indicates that the effects of lactylation on OA chondrocytes can be broadly divided into pathological, pro-degenerative outputs and adaptive, chondroprotective responses under specific conditions. These divergent effects are not determined simply by lactate concentration or global lactylation levels. Rather, they depend on whether lactylation occurs on histones or non-histone proteins, the specific modification site and protein substrate involved, the downstream functional network engaged, and the metabolic and redox state of the chondrocyte at the time of modification (Lu et al., 2026; Dong et al., 2026; Lan et al., 2025).

Pathological lactylation can disrupt cartilage homeostasis at both the protein-functional and transcriptional levels. UGDH K6 lactylation exemplifies a mechanism through which non-histone lactylation directly compromises matrix maintenance. This modification reduces UGDH enzymatic activity and restricts glycosaminoglycan synthesis. It also alters the nucleocytoplasmic distribution of UGDH and weakens its interaction with STAT1, thereby relieving repression of MAP3K8 transcription and enhancing MAPK-dependent catabolic signaling. The consequence is not merely an increase in individual matrix-degrading molecules, but a simultaneous decline in both matrix synthesis and the capacity of chondrocytes to maintain their surrounding extracellular matrix (Lan et al., 2025). By contrast, the FSTL1–H3K18la axis illustrates how histone lactylation can reshape chondrocyte identity. FSTL1-driven glycolysis and lactate accumulation increase H3K18la enrichment at fibrosis-related gene loci and promote their transcription, progressively shifting chondrocytes away from the mature hyaline cartilage phenotype toward a fibrocartilage-like state and aggravating cartilage fibrosis in experimental OA (Lu et al., 2026). Together, these studies show that pathological lactylation can convert transient metabolic stress into persistent matrix damage and phenotypic drift through two distinct mechanisms: loss of non-histone protein function and chromatin-based transcriptional reprogramming.

In contrast to these pro-degenerative outputs, H3K56la exerts an adaptive and chondroprotective effect under specific post-traumatic and redox conditions. Oxidative stress reduces nuclear lactylation in chondrocytes, whereas a relatively reducing environment promotes lactate uptake and restores H3K56la. This restoration enhances HIF-1α expression and its transcriptional regulation of the COL2A1 promoter, increases type II collagen synthesis, and reduces oxidative stress-induced chondrocyte apoptosis. Activation of this pathway also ameliorates cartilage pathology in an anterior cruciate ligament transection model of post-traumatic OA. These findings indicate that lactate entering chondrocytes is not inevitably translated into catabolism or cell death. In cells that retain reparative capacity and exist within an appropriate redox environment, H3K56la may instead direct lactate-derived signals toward matrix anabolism and stress protection (Dong et al., 2026).

UGDH K6 lactylation, the FSTL1–H3K18la axis, and the H3K56la–HIF-1α–COL2A1 pathway should therefore not be viewed as isolated or genuinely contradictory mechanisms. Rather, they collectively reveal a central principle of lactylation-dependent chondrocyte fate regulation. Lactate represents a shared metabolic input, but the modified site and protein substrate determine the regulatory level at which this signal operates. The chromatin state, redox environment, and disease stage of the cell then further determine whether the signal is amplified into matrix destruction, fibrosis, and increased susceptibility to cell death, or redirected toward matrix synthesis and stress adaptation. The H4K12la–TRIM29 pathway in chondrocyte senescence and the H3K18la–ACSL4 pathway in ferroptosis further expand the spectrum of pathological lactylation outputs (Zuo et al., 2026). Lactylation is therefore better understood as a site-, substrate-, and context-dependent metabolic “fate code” rather than a uniformly pro-degenerative signal.

This bidirectional framework also changes how lactylation-targeted intervention should be considered. A global reduction in lactylation does not necessarily translate into therapeutic benefit, because broadly suppressing lactate production or lactylation-writing mechanisms may attenuate pathological responses such as UGDH K6 lactylation and H3K18la while simultaneously disrupting potentially adaptive responses mediated by H3K56la. Future studies should therefore move beyond measurements of global lactylation and instead identify disease-driving modification sites, target proteins, and cell states, while defining the temporal windows in which distinct lactylation programs operate across OA etiologies and stages. Such knowledge will be essential for selectively blocking pathological lactylation circuits while preserving the metabolic adaptations required for chondrocyte survival and repair.

## 5 Lactate-centered metabolic crosstalk in the inflammatory joint microenvironment

The studies discussed above show that lactylation can convert lactate accumulation within chondrocytes into phenotypic drift, cellular senescence, and altered susceptibility to cell death. OA, however, is not driven by chondrocyte-autonomous dysfunction alone. Cartilage, synovium, intra-articular immune cells, and subchondral bone occupy a relatively enclosed metabolic space. Lactate produced by any of these cells may diffuse through synovial fluid or cross cell membranes through monocarboxylate transporters, changing the metabolic pressure on neighboring cells. Synovial fluid from patients with OA contains more lactate, together with increased global protein lactylation in chondrocytes (Lan et al., 2025). Lactate may therefore have two roles. Extracellularly, it is an exchangeable metabolite that supports communication between tissues. After entering a cell, it may be translated through lactylation into more persistent changes in protein function and transcription. Chondrocyte lactylation should thus be considered within the dynamic network of lactate production, transport, and use across the entire joint, rather than as an isolated intracellular event.

The most direct evidence from OA synovium currently concerns lactate itself rather than lactylation. Huang et al. found that elevated lactate aggravated synovitis in OA rats and in an intra-articular lactate injection model. In human OA fibroblast-like synoviocytes, exogenous lactate upregulated arginase 2 (ARG2) and induced G1/S arrest, ROS accumulation, SA-β-gal activity, and SASP expression. ARG2 knockdown, mTOR inhibition with rapamycin, or PI3K inhibition with LY294002 attenuated lactate-induced senescence. The ARG2-mTOR/S6K1 axis therefore appears to mediate the effect of lactate on synovial cell state (Huang et al., 2024). Lactate not only affects chondrocytes, but can also drive fibroblast-like synoviocytes toward a senescent phenotype that continuously secretes inflammatory mediators and prolongs synovitis. This study did not assess histone or nonhistone lactylation, however, so ARG2 upregulation cannot be attributed directly to lactylation. The evidence establishes synovial cells as lactate-responsive cells, while the involvement of site-specific lactylation in their senescence remains to be tested.

Synovial cells do not merely sense lactate. By reprogramming immune-cell metabolism, they may also expand the local lactate pool. Liu et al. found that macrophages took up exosomes from inflammatory fibroblast-like synoviocytes. This increased HIF-1α, GLUT1, HK2, pyruvate kinase M2 (PKM2), and LDHA, along with extracellular acidification, glucose consumption, and lactate release. It also increased inflammatory markers, including inducible nitric oxide synthase (iNOS), cluster of differentiation 86 (CD86), NLR family pyrin domain-containing 3 (NLRP3), IL-1β, IL-6, and TNF-α. Genetic HIF-1α silencing, pharmacological inhibition with PX-478, or glycolytic blockade with 2-deoxy-D-glucose suppressed the macrophage inflammatory phenotype. Intra-articular injection of inflammatory exosomes aggravated synovitis and cartilage degeneration in mice (Liu B. et al., 2024). Cell-cell communication within the OA synovium can therefore directly reshape lactate-producing capacity. This observation raises an important question: does lactate generated by glycolytic macrophages alter the lactylation profile of macrophages, synovial cells, or neighboring chondrocytes, in addition to its established metabolic and inflammatory effects? OA studies have not yet answered this question, so the macrophage inflammatory phenotype should not be described as a downstream effect of lactylation.

Cartilage injury and synovial metabolic activation may therefore amplify each other. Matrix fragments and damage-associated molecules released from degenerating cartilage stimulate synovial cells and macrophages. For example, the damage-associated calcium-binding protein complex S100A8/A9 acts as an alarmin and remains elevated in the synovium of experimental OA with pronounced inflammation. S100A9 deficiency reduced synovial activation by approximately 62% and cartilage damage by 45%–73%. Damage alarmins are therefore not merely by-products of cartilage degeneration, but help sustain synovial inflammation (van Lent et al., 2012). Conversely, S100A8/A9 stimulation of human OA chondrocytes increased the expression of the matrix-degrading enzymes MMP1, MMP3, MMP9, and MMP13, together with the inflammatory cytokine and chemokine mediators IL-6, IL-8, and MCP-1. These effects were mediated through Toll-like receptor 4 (TLR4) signaling and were accompanied by reduced expression of the cartilage matrix markers ACAN and COL2A1. Inflammatory signals from synovium and synovial fluid can thus impair cartilage homeostasis (Schelbergen et al., 2012). Recent studies further show that extracellular vesicles released by inflammatory or senescent synovial cells can simultaneously disrupt chondrocyte matrix homeostasis, promote inflammatory macrophage phenotypes, and inhibit chondrogenic differentiation of mesenchymal stem cells (Liu et al., 2025). Together, these findings support substantial bidirectional communication between cartilage and synovium.

Lactate may not act in the same direction in every joint cell. Experiments in rheumatoid arthritis synovium showed that the same lactate concentration enhanced migration, glycolysis, and IL-6 secretion in synovial fibroblasts, but reduced glycolysis, migration, and IL-6 release in macrophages. These distinct responses were associated with differences in MCT1 and MCT4 expression (Pucino et al., 2023). Although these findings cannot be directly extrapolated to OA, they show that elevated local lactate does not imply a uniform proinflammatory response across joint cells. Its effect may depend on concentration, transport direction, cellular redox state, and the pre-existing chromatin landscape. If lactylation is involved, different cell types and lysine sites may likewise produce opposing transcriptional outcomes.

In contrast to the unproven causal role of lactylation in synovium, preliminary direct evidence is available for subchondral bone. Xu et al. performed proteomic and lactylomic analyses of OA subchondral bone. They identified 668 lactylated proteins and found that H4K12la was markedly elevated. The change was strongly cell-specific: H4K12la increased in osteoblasts but decreased in osteoclasts, although osteoclasts produced more lactate. Exogenous lactate increased H4K12la and osteogenic markers in osteoblasts, whereas p300 inhibition or blockade of MCT1-mediated lactate uptake attenuated these effects. The MCT1/MCT4 inhibitor syrosingopine also reduced subchondral bone sclerosis in rats after anterior cruciate ligament transection (Xu et al., 2026). These results support a model of intercellular metabolic coupling. Lactate produced by osteoclasts is transported into osteoblasts through MCTs, activates an osteogenic program through H4K12la, and ultimately promotes abnormal subchondral bone sclerosis in OA. This study was the first to place lactate transport, cell-specific histone lactylation, and OA bone remodeling in one causal chain. The evidence still comes mainly from a single study, and the direct H4K12la target genes and their temporal relationship with cartilage damage remain unknown.

**FIGURE 4:**
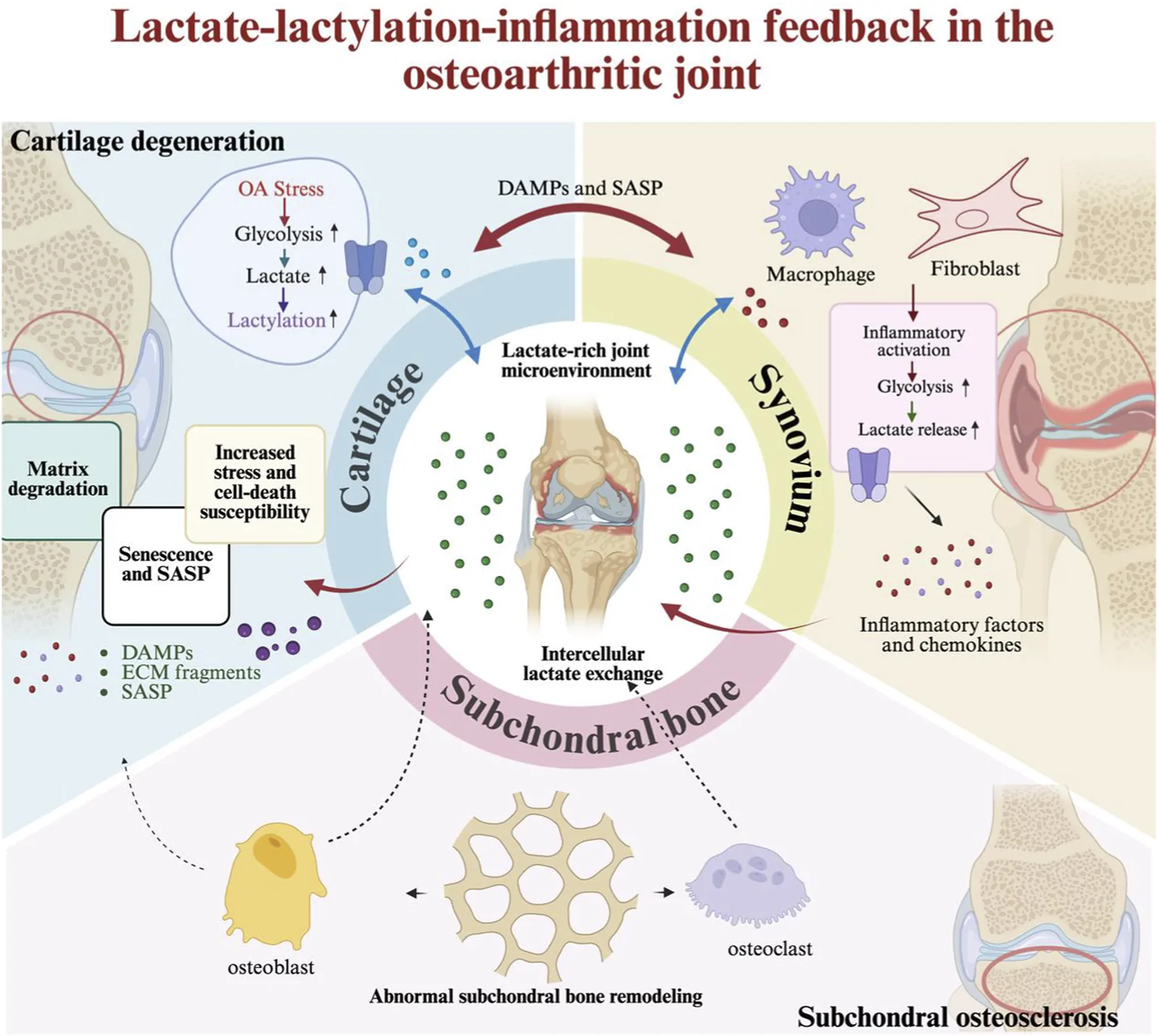
Lactate–lactylation–inflammation feedback in the osteoarthritic joint:Osteoarthritic stress promotes glycolytic reprogramming and lactate accumulation in articular cartilage, leading to enhanced lactylation, matrix degradation, senescence-associated secretory phenotype (SASP), and increased cellular stress or death susceptibility. Damaged cartilage releases DAMPs, extracellular matrix fragments, and SASP factors that amplify synovial inflammation. In the synovium, activated macrophages and fibroblasts undergo metabolic reprogramming, increase glycolytic activity and lactate release, and produce inflammatory mediators and chemokines. Bidirectional lactate exchange between cartilage and synovium further sustains a lactate-rich joint microenvironment, thereby reinforcing metabolic and inflammatory signaling across joint tissues. Abnormal subchondral bone remodeling and osteosclerosis may additionally contribute to this inter-tissue communication. Together, these reciprocal interactions establish a self-reinforcing lactate–lactylation–inflammation feedback loop that may promote the persistence and progression of osteoarthritis. Created with BioRender.com. Publication license number: ZV2A3P888N.

Taken together, these findings suggest a multi-tissue regulatory network in the OA joint in which lactate serves as a shared metabolite and lactylation translates that information within cells. Evidence shows that lactate can induce senescence in fibroblast-like synoviocytes, synovial signals can enhance macrophage glycolysis, and lactate can pass from osteoclasts to osteoblasts and induce H4K12la in subchondral bone. By contrast, a closed cartilage-synovium loop directly mediated by lactylation has not been established. The most reasonable current model does not place lactylation upstream of every inflammatory response in the joint. Instead, lactylation is a cell- and site-selective metabolic decoding mechanism. A shared lactate environment provides a similar metabolic input to different joint cells, but their transport capacity, enzymatic machinery, and chromatin context determine whether that input becomes inflammation, senescence, matrix degradation, or bone remodeling. This interpretation preserves the potential of the lactate-lactylation axis to explain whole-joint disease without presenting unverified tissue connections as established mechanisms (Figure 4).

## 6 Therapeutic implications and future perspectives

The lactate-lactylation axis provides a therapeutic level between the upstream metabolic environment and downstream degenerative phenotypes in OA. Conventional anti-inflammatory or anticatabolic treatments mainly target inflammatory mediators and matrix-degradation products after they have formed. Lactylation acts before these effector molecules are expressed by converting lactate accumulation into changes in gene transcription and protein function. Targeting lactylation may therefore affect not only one inflammatory mediator, but also matrix synthesis, phenotypic drift, senescence, and susceptibility to cell death in chondrocytes. Because several pathological processes jointly drive OA, a regulatory node at the interface of metabolism and cell phenotype may offer broader disease-modifying potential than a single terminal effector.

Animal studies provide initial evidence that this process is amenable to intervention. Inhibition of lactate dehydrogenase (LDH) -dependent lactate production, blockade of MCT-mediated lactate transport, p300 inhibition, and enhancement of SIRT1 activity can each reduce selected pathological lactylation events and improve cartilage degeneration, cellular senescence, or abnormal subchondral bone remodeling in experimental OA (Liu et al., 2025; Pucino et al., 2023; Xu et al., 2026). These findings do not yet identify the most clinically suitable strategy, but they show that lactate accumulation and downstream lactylation are not irreversible disease endpoints. The interventions act at distinct levels, including lactate production, inter-tissue transport, and the lactylation effector stage. This diversity may allow therapy to be matched to the affected tissue and stage of OA.

Recent work has begun to combine lactylation biology with local delivery and tissue repair. Qian et al. developed an adhesive hydrogel containing a positively charged lactate oxidase (LOX) nanodrug. A single intra-articular administration sustained release for approximately 20 days. By regulating chondrocyte glycolysis and lactate production, the system altered inflammation-related histone lactylation. In animal models, it reduced synovitis, promoted cartilage-defect repair, and delayed OA progression. Experiments with chondrocyte-specific Ldha deletion further supported a role for lactate in the therapeutic effect (Qian et al., 2026). Lactylation-directed treatment may therefore not require prolonged systemic use of metabolic inhibitors. It may instead restore local metabolic homeostasis while extending drug retention within the joint. Combining metabolic regulation with local biomaterial delivery may be a more practical translational approach for articular cartilage, which is poorly vascularized, has a dense matrix, and clears drugs rapidly.

Another strategy is to interrupt lactylation programs driven by specific disease signals. FSTL1 increases glycolysis and H3K18la in chondrocytes and promotes fibrosis-related gene expression. FSTL1 silencing, PI3K/mTOR inhibition, or reduced lactate production lowered H3K18la and cartilage fibrosis, while exogenous lactate partly restored the phenotype (Lu et al., 2026). Therapy may therefore target upstream signals that cause pathological lactylation without suppressing all lactate metabolism. Similarly, downstream effects linked to TRIM29, ACSL4, TPI1, and lactylated UGDH offer more specific targets for senescence, ferroptosis, glycolytic positive feedback, or impaired matrix metabolism. As pathogenic sites and their targets become clearer, lactylation-directed therapy may shift from broad metabolic suppression toward blockade of defined pathological circuits.

Current evidence suggests two broad therapeutic paths. The first is to correct the abnormal lactate environment within the joint by limiting pathological glycolysis, reducing excess lactate production, or blocking harmful intercellular lactate transfer. This broad approach may suit OA phenotypes with markedly elevated lactate and metabolic dysfunction across several tissues. The second is to target lactylation effectors directly, including specific sites, target proteins, or downstream transcriptional programs. This approach should be more selective and may suit cell populations with an established pathogenic lactylation circuit. The two paths are not mutually exclusive. A local delivery system could first reduce the lactate burden in the joint cavity and then use a targeted molecule to restrict pathological lactylation in a specific cell type, thereby limiting interference with normal cartilage metabolism.

Lactylation-directed therapy faces two main limitations. First, lactylation is strongly site- and context-dependent. H3K18la, H4K12la, and some nonhistone lactylation events are associated with OA degeneration, whereas H3K56la may maintain HIF-1α-dependent COL2A1 expression in certain post-traumatic settings. A decrease in global lactylation does not necessarily indicate therapeutic benefit. Future studies must assess individual sites, target cells, and disease stages. Second, available modulators of LDH, MCTs, p300, and SIRT1 affect metabolic or epigenetic processes beyond lactylation. Any cartilage-protective effect after treatment must therefore be linked to lactylation through site-directed mutations, target-gene rescue, and cell-specific genetic models. These limitations preclude a direct move from correlational findings to broad pharmacological inhibition of lactylation.

Future research should establish pathological lactylation maps that can guide treatment selection. Comparing lactate sources, key lactylation sites, and their target genes across OA subtypes and stages may identify patients whose disease is truly dominated by dysregulation of the lactate-lactylation axis. Lactate in synovial fluid, lactylated proteins in extracellular vesicles, and specific lactylation-related transcriptional signatures may support patient stratification and pharmacodynamic monitoring. Cartilage-penetrating carriers, injectable hydrogels, and cell-selective delivery could then restrict intervention to the appropriate tissue, cell type, and time window.

Overall, the lactate–lactylation axis provides a new mechanistic framework for understanding how metabolic reprogramming is translated into phenotypic and fate changes in OA chondrocytes. Histone and non-histone lactylation can regulate matrix metabolism, fibrosis, cellular senescence, and susceptibility to cell death at distinct levels, and may also contribute to metabolic crosstalk among cartilage, synovium, and subchondral bone. Importantly, lactylation is not a uniformly pro-degenerative signal. UGDH K6 lactylation and FSTL1-driven H3K18la represent pathological outputs that impair matrix maintenance and promote a fibrotic phenotype, respectively. By contrast, the H3K56la–HIF-1α–COL2A1 axis indicates that, under specific post-traumatic and redox conditions, lactylation can support type II collagen synthesis and enhance chondrocyte adaptation to stress. Thus, the biological effects of lactylation are determined not by the overall level of this modification, but by the modified site, protein substrate, cellular state, and disease stage. Future studies should move beyond global changes in lactylation and focus on cell-, site-, and stage-specific mechanisms supported by causal evidence. Combined with local delivery strategies and metabolic phenotype-based stratification, this approach may enable the development of disease-modifying therapies that selectively disrupt pathological lactylation programs.
